# Kinetics of Cardiac Output at the Onset of Exercise in Precapillary Pulmonary Hypertension

**DOI:** 10.1155/2016/6050193

**Published:** 2016-11-20

**Authors:** Frédéric Lador, Aurélien Bringard, Samir Bengueddache, Guido Ferretti, Karim Bendjelid, Paola M. Soccal, Stéphane Noble, Maurice Beghetti, Denis Chemla, Philippe Hervé, Olivier Sitbon

**Affiliations:** ^1^Service de Pneumologie, Programme Hypertension Pulmonaire, Hôpitaux Universitaires de Genève, Genève, Switzerland; ^2^Faculté de Médecine, Université de Genève, Genève, Switzerland; ^3^Département d'Anesthésiologie, Pharmacologie et Soins Intensifs, Faculté de Médecine, Université de Genève, Genève, Switzerland; ^4^Département de Médecine Moléculaire et Translationnelle, Université de Brescia, Brescia, Italy; ^5^Service de Cardiologie, Hôpitaux Universitaires de Genève, Genève, Switzerland; ^6^Unité de Cardiopédiatrie, Programme Hypertension Pulmonaire, Hôpitaux Universitaires de Genève, Genève, Switzerland; ^7^Université Paris-Sud, Le Kremlin-Bicêtre, France; ^8^INSERM UMR_S999, LabEx LERMIT, Centre Chirurgical Marie Lannelongue, Le Plessis Robinson, France; ^9^AP-HP, Centre de Référence de l'Hypertension Pulmonaire Sévère, Département Hospitalo-Universitaire (DHU) Thorax Innovation (TORINO), Service de Pneumologie, Hôpital de Bicêtre, Le Kremlin-Bicêtre, France

## Abstract

*Purpose.* Cardiac output (CO) is a cornerstone parameter in precapillary pulmonary hypertension (PH). The Modelflow (MF) method offers a reliable noninvasive determination of its beat-by-beat changes. So MF allows exploration of CO adjustment with the best temporal resolution.* Methods.* Fifteen subjects (5 PH patients, 10 healthy controls) performed a submaximal supine exercise on a cycle ergometer after 5 min of rest. CO was continuously determined by MF (CO_MF_). Kinetics of heart rate (HR), stroke volume (SV), and CO were determined with 3 monoexponential models.* Results.* In PH patients, we observed a sudden and transitory drop of SV upon exercise onset. This implied a transitory drop of CO whose adjustment to a new steady state depended on HR increase. The kinetics of HR and CO for PH patients was slower than that of controls for all models and for SV in model 1. SV kinetics was faster for PH patients in models 2 and 3.* Conclusion.* This is the first description of beat-by-beat cardiovascular adjustments upon exercise onset in PH. The kinetics of HR and CO appeared slower than those of healthy controls and there was a transitory drop of CO upon exercise onset in PH due to a sudden drop of SV.

## 1. Introduction

Precapillary pulmonary hypertension (PH) is a hemodynamic condition due to increased pulmonary vascular resistances (PVR) leading to progressive right ventricular failure [1]. It is associated with progressive exercise intolerance as compared to healthy subjects, due to the inability of the right ventricle (RV) to cope with increased afterload [1]. This may also be due to a compression of the left ventricle (LV) by the dilated RV, thus limiting the expected physiological increase of stroke volume (SV) at the onset of exercise [2]. Changes in the adrenergic pathways may be also involved, implying a tapered contractility and chronotropic response of RV [3]. All these mechanisms contribute to altering cardiac output (CO) response to exercise and help understand the prognostic value of several RV and LV physiological parameters [4–6].

Recommended and developing techniques dedicated to CO measurement, like thermodilution (TD), allow only steady-state determination of this parameter [7–9]. Yet, it has been recently shown that the beat-by-beat and noninvasive assessment of CO based on the arterial pulse pressure wave analysis called Modelflow (MF) [10] is a reliable and accurate procedure in precapillary PH [11] and healthy subjects [12]. Thus, it allows description of the CO adjustment during metabolic transients with the best possible temporal resolution. We hypothesized that the beat-by-beat description of CO kinetics upon exercise onset in precapillary PH would be slower than those of healthy subjects.

## 2. Methods

### 2.1. Study Population

Five patients with precapillary PH (4 with pulmonary arterial hypertension (PAH) and 1 with chronic thromboembolic PH (CTEPH) with residual PH after pulmonary endarterectomy) and 10 healthy control subjects took part in the experiments (Table 1). All subjects were preliminarily informed of all procedures and risks associated with the experimental testing. Informed consent was obtained from each volunteer, who was aware of the right of withdrawing from the study at any time without jeopardy. The study was conducted in accordance with the Declaration of Helsinki. The protocol was approved by the institutional ethical committee.

### 2.2. Right Heart Catheterization (RHC)

Invasive hemodynamic evaluation was carried out in PH patients in a recumbent position as previously described [11] and included measures of pulmonary artery mean pressure (mPAP), pulmonary artery wedge pressure (PAWP), right atrial pressure (RAP), CO by TD (CO_TD_), PVR calculated as the transpulmonary gradient (mPAP − PAWP) divided by CO_TD_, and systemic vascular resistances (SVR) as mean systemic arterial pressure (MAP) divided by CO determined by MF (CO_MF_), on the supposition that the RAP can be excluded from its calculation [13, 14].

### 2.3. Cardiac Output by Thermodilution and by Modelflow®

CO_TD_ and CO_MF_ were determined as previously proposed [11] by injection of 10 mL of iced-cold sterile, isotonic glucosaline solution through the proximal catheter's lumen and from continuous noninvasive recording of arterial pulse pressure profiles by a Portapres® system (TNO-TPD, Amsterdam, Netherlands), respectively. Mean CO_MF_ was calculated as the mean beat-by-beat value obtained over 1 min at steady state, at rest, and after 2-minute exercise.

### 2.4. Exercise Hemodynamics

Hemodynamic evaluation was realized in a recumbent position by means of an electromagnetically braked cycle ergometer (Cycline 100; Tecmachine, Andrezieux-Boutheon, France, or Ergoselect 400, Ergoline GmbH, Bitz, Germany) adapted to the experimental table. Before exercising, the subjects feet were installed on the pedals (raised legs position). A 5 min delay was observed before recording to ensure hemodynamic stabilization at a new steady state. Then, the patients pedaled at 60 rpm at a workload of 20 W (PH patients) or 50 W (healthy controls) for 5 min. CO_TD_ was determined in PH patients at rest and during the last minute of exercise. Workload was determined in order to correspond for each group to the same mean proportional increase in the heart rate (HR) reserve. All measures were obtained at steady HR. The pedaling frequency was recorded, and its sudden increase at the exercise onset was used as marker to identify precisely the start of exercise. The electromechanical characteristics of the ergometer were such as to permit workload application in less than 50 ms.

### 2.5. Data Treatment

Before the analysis of on-kinetics, the beat-by-beat HR, SV, MAP, and CO values of all subjects were time aligned, by setting the time of exercise start as time zero. As proposed by Bringard and coworkers [13], no interpolation was performed and the analysis was performed on the beat-by-beat data. Based on previous published findings [2, 15], the kinetics of SV, CO, and HR were described with a monoexponential model firstly proposed by Barstow and Molé [16], whereby a flow increase with a time delay reflects the circulation time from active muscles to the lungs. The equation was

(1) $$\begin{array}{c} F_{\left(t\right)}=b+a\theta \left(\;t-d\;\right)\left(\;1-e^{-\left(t-d\right)/\tau }\;\right), \end{array}$$

where *t* is time. The parameter *b* represents resting value. The parameter *a*_1_ is the amplitude of the exponential, and the parameter *τ*_1_ is the time constant. The time *d*_1_ is the time delay of the exponential. The function *θ* is the Heaviside function (*θ* (*t*) = 0 if *t* < 0 and *θ* (*t*) = 1 if *t* ≥ 0). Mean response time (MRT) is defined by *τ*_1_ + *d*_1_.

**Figure 1:**
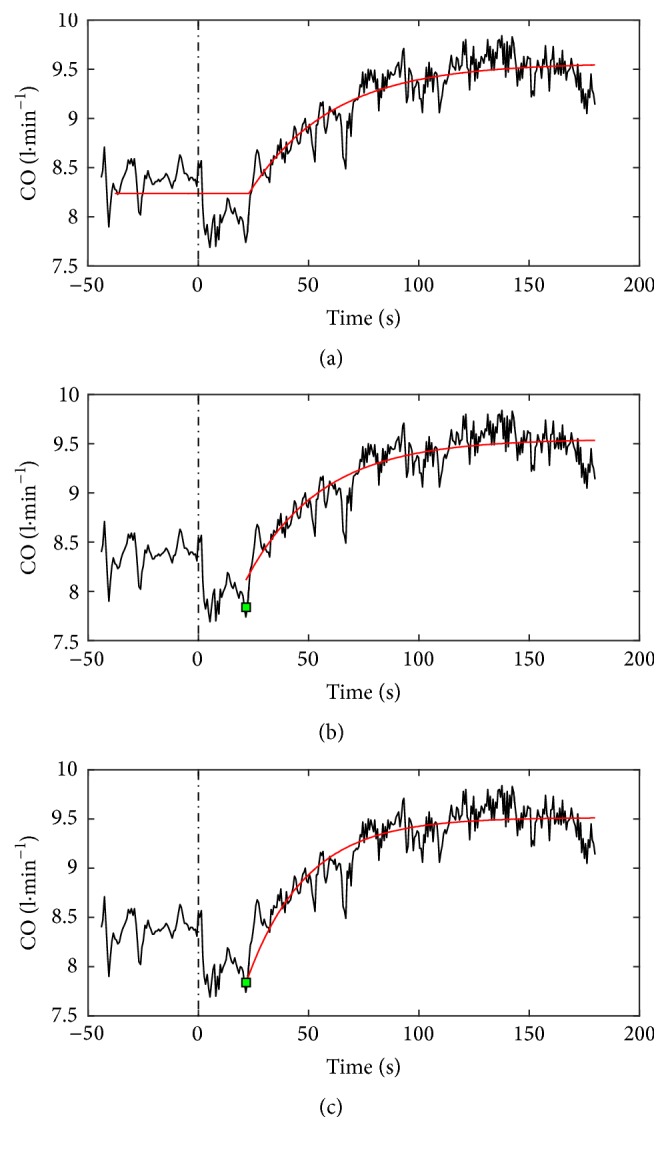
Graphical representation of the 3 models used for CO data analysis. CO, cardiac output; time 0 (black dotted line) corresponds to exercise onset; the red line in panels (a), (b), and (c) corresponds to the 3 models description; the green point in panels (b) and (c) corresponds to the minimal CO output (CO_min_) determined for models 2 and 3, respectively. CO data of the PH patients were temporally aligned and superimposed. Analysis was performed individually for all subjects.

Since PH patients exhibited a decrease of SV and CO during the first 10–20 s after initiation of exercise, two alternative models, both also based on (1), are proposed for CO and SV analysis. These 2 alternative models require detection of the minimum value of the studied parameter (SV_min_ and CO_min_), along with the corresponding time (*t*_SVmin_ or *t*_COmin_). To avoid detection of coincidental minimum due to the presence of noise, detection of CO_min_ or SV_min_ was performed on smoothed data, using Savitzky and Golay [17] smoothing filter of the beat-by-beat data. It is noteworthy that smoothed data are only used for the detection of CO_min_ or SV_min_, and the modelling was performed on the original beat-by-beat CO and SV data. Specifically, in the second model, after detection of CO_min_ or SV_min_ and *t*_COmin_ or *t*_SVmin_, the same model as for model 1 (1) was applied on the beat-by-beat data, starting from *t*_min_. It is noteworthy that when using regression method to fit physiological data like ours, all parameters of the equation model used interfere together. For instance, in (1), the time constant (*τ*_1_) is influenced by the rest data and parameter *b*. Although model 1 takes into account rest data for the fitting, in model 2, by omitting the rest data and starting from *t*_min_, the beats in the early increase of the kinetics lose weight in the least square regression compared to beats in the steady-state region of the curve. Indeed, with CO_min_ and SV_min_ being both a single value, compared to the parameter *b* in (1) being computed on several beats, the fitting would be theoretically less robust when passing through the early beats after CO_min_ or SV_min_, as represented by the residuals. To cope with this issue, we proposed a third method (model 3), in which the model was forced to pass through CO_min_ or SV_min_ data point and the parameters *b* and *d*_1_ of (1) were, respectively, replaced by CO_min_ or SV_min_ and *t*_COmin_ or *t*_SVmin_. A graphical representation of the 3 models applied on merged CO data of the PH patients group is proposed in Figure 1. The same analysis was performed on the control group. In order to adapt workloads intensity for each group, resting and theoretical maximal HR (determined as 220 − age) were used to calculate, for each patient, HR reserve, considering the mean resting value as 0% and the theoretical maximal value as 100%.

### 2.6. Statistics

The SV, CO, and HR series were fitted by a weighted nonlinear least squares procedure [18–20] implemented under MATLAB (version 7.13.0.564, MathWorks, Natick, MA, USA). Initial guesses of the parameters of the model were entered after visual inspection of the data. Equation (1) was used as the fitting model. In other words, the estimated model parameters *b*, *a*_1_, *τ*_1_, and *d*_1_ were obtained by minimizing the squared difference between the model function and the experimental data. Constraints were set as parameters *b*, *a*_1_, *τ*_1_, and *d*_1_ could not be negative. Time 0 was defined as the instant at which exercise started. In order to facilitate comparison, time course of HR, SV, and CO was also determined as relative value, with 0% and 100% being resting and steady-state exercise values, respectively.

## 3. Results

**Figure 2:**
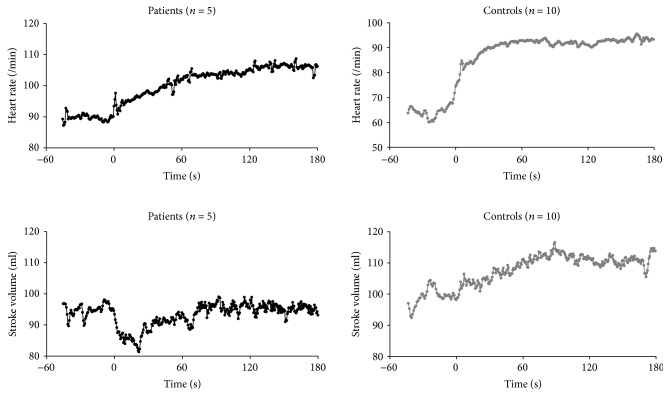
Time course of beat-by-beat heart rate (HR) and stroke volume (SV) upon exercise onset. Time 0 corresponds to exercise start. Data were temporally aligned and superimposed for both groups.

**Figure 3:**
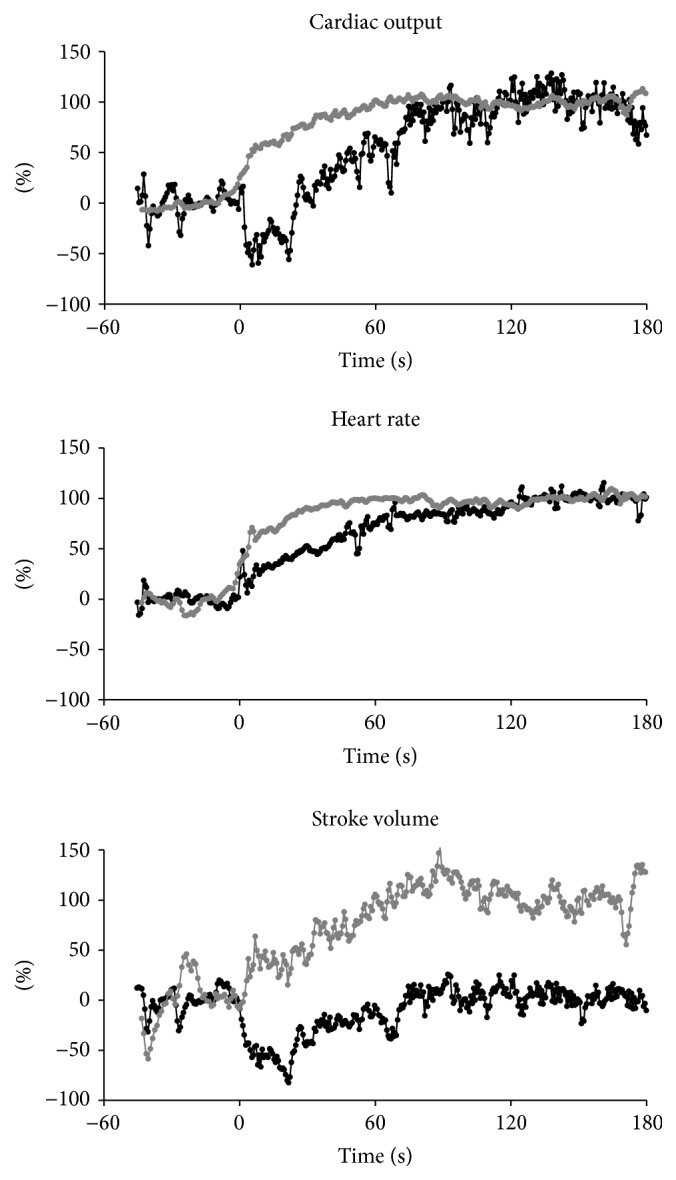
Time course of beat-by-beat heart rate (HR), stroke volume (SV), and cardiac output (CO) upon the onset of exercise. Data are superimposed for all PH patients (black, *n* = 5) and control subjects (grey, *n* = 10) and represented as relative values from 0 (baseline) to 100% (steady-state exercise). Time 0 corresponds to exercise start. Data were temporally aligned and superimposed for both groups.

**Figure 4:**
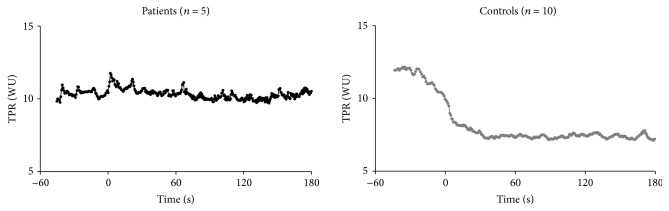
Time course of systemic vascular resistances (SVR) upon exercise onset. Time 0 corresponds to exercise start. Data were temporally aligned and superimposed for both groups.

The clinical and hemodynamic data at baseline and steady-state exercise are shown in Tables 1 and 2. The time courses of HR and SV upon the onset of exercise are shown in Figure 2 for PH patients and control subjects. For the control subjects, these parameters increased at exercise onset to reach a new steady state. For the PH patients, we observe the same phenomenon for HR but such was not the case for SV. Here, we observed a sudden drop that lasted for 10 to 20 s before reaching the baseline level with an increase at steady-state exercise as compared to resting values. Time courses of HR, SV, and CO upon exercise onset for PH patients and control subjects are represented in Figure 3. The mean HR increase corresponded to 24.3% ±5.1 and 24.6% ±26.0 of the theoretical HR reserve for healthy controls and PH patients, respectively. At the onset of exercise, CO increased for the control group to reach a new steady state. For the PH patients, CO decreased over 3 to 5 beats where it stabilized during 20 s before increasing and reaching a new steady level, higher than its resting value. The time course of SVR upon exercise onset is represented in Figure 4. For the control subjects, we observed a sudden drop at the onset of exercise but these changes were not visible for the PH patients.

The characteristic parameters describing the HR, SV, and CO kinetics according to the 3 theoretical models upon exercise onset are presented in Table 3. For each parameter, we observed a steady-state value higher than that of baseline (rest) with the 3 different models, except for SV assessed by method 1 in the PH patients where SV remained stable between baseline (90.6 ± 32.1 mL) and steady state (97.6 ± 38.8 mL). MRT for HR and CO was longer in PH patients as compared to control subjects regardless of the model used. For SV, the overall kinetics assessed by model 1 was clearly slower in PH patients, as evidenced by a MRT of 133.5 ± 13.7 s, much longer than that of control subjects (20.2 ± 13.4 s). Here, model 1 provided inaccurate kinetics description, due to its inability to detect sudden drop of SV at the onset of exercise. Models 2 and 3, considering analysis upon minimal SV detection, revealed faster kinetics for SV in PH patients than in control subjects. SV Analysis with model 2 was not possible for 2 control subjects, due to the inability of the model to detect SV_min_, providing aberrant kinetics characteristics.

## 4. Discussion

In this pilot study, we described for the first time the beat-by-beat kinetics of CO at exercise onset in PH patients. The overall kinetics of cardiovascular adjustment in response to supine exercise appeared to be slower in PH patients than in control subjects, in agreement with the tested hypothesis. Moreover, there was a decrease in CO at the onset of exercise that was due to a transitory drop in SV. It is noteworthy that this pattern of response to exercise was recorded in all the 5 PH patients under study. Moreover, our results suggest that there was no significant increase of SV during exercise and that virtually all the CO increase in response to exercise could be attributed to the HR changes. On the other hand, we observed that the HR kinetics was slower in PH patients than that of healthy controls, whatever the model used for the data analysis. In fact, HR response was not only limited in its amplitude, as previously demonstrated [3] (the so-called impaired chronotropic response in PAH) but also in its kinetics, which was systematically slower than that of healthy subjects. This is in line with previously published work that demonstrated a close relationship between HR and mPAP in exercising patients with severe precapillary PH [21]. The close relationship between CO response and kinetics of oxygen uptake (VO_2_) upon exercise onset in healthy subjects [2, 14, 22–25] and in heart failure [26, 27] is also well known. It was also suggested in pulmonary vascular diseases [28, 29]. In agreement with the present results, reported VO_2_ kinetics is blunted in this group of patients. With kinetics of VO_2_ being reported and currently accepted to be described as an exponential [30–33], any change would be best detected at exercise onset. To the best of our knowledge, however, no study described or indirectly suggested a transitory decrease of CO at the early onset of exercise in these conditions. This may be related to the lower sampling frequency during the initial cardiodynamic phase, limited by breathing pattern. Indeed, the higher frequency of cardiovascular variables assessed on a beat-by-beat basis together with their inherent excellent signal-to-noise ratio compared to pulmonary VO_2_ allows robust kinetics analysis and may have permitted the present findings.

### 4.1. About SV Kinetics

It is possible that the sudden increases in venous return may have induced, at least transiently, an increase in the RV end-diastolic volume and have worsened the RV dysfunction at the onset of exercise. Accordingly, a transitory fall of LV preload associated with an exaggerated compression of the LV by the RV will lead to a global decrease of LV end-diastolic volume and transient drop in SV that is not counterbalanced by the HR increases. The resulting effect is a sudden drop in CO. It may also be that the transitory decreased SV may be an additional marker of hemodynamic transitional RV inability to accept an additional burden of venous return and, therefore, consequently, a marker of poor outcome. This could be the case, considering the severity of the hemodynamic impairment of the PH patients included in this study. Alternatively, it is also possible that dysfunctional RV, at the onset of exercise, becomes unable to cope with an increased afterload (mPAP). This, together with a retarded or inoperative inotropic effect (in response to increased afterload), may have led to the observed transitory SV reduction. It is also noteworthy that SV kinetics determined by models 2 and 3 were faster for PH patients than that of control subjects. This is also an argument toward a decreased volume reserve in PH and a new steady volume attained earlier.

### 4.2. About Exercise and PH

Exercise hemodynamics is becoming of primary importance in the evaluation of pulmonary vascular diseases. In fact, it has been well demonstrated to better describe the real resistive properties of pulmonary vascular bed [34, 35]. Thereby, it may be a decisive help for revealing pulmonary vascular disease undetected by resting measurements [36–40], assessing PAH severity or driving its therapeutic approach [41]. As suggested in the present study, exercise exploration could provide new information on the mechanisms of cardiodynamic adjustments in diseases. It may also help unmasking postcapillary PH that may occur in patients with heart failure with preserved ejection fraction [42]. Moreover, recently published studies proposed new criteria for diagnosis of exercise pulmonary hypertension [36, 37] or demonstrated prognostic role of exercise hemodynamics [43], reinforcing the interest of hemodynamic exploration during exercise. As recommended by current guidelines [44], rehabilitation programs are now part of the standard care for PH patients. Here, availability of inexpensive and reliable noninvasive CO monitoring may be desirable and help distinguish the hemodynamic determinants of exercise intolerance in PH patients.

### 4.3. Limitations of the Present Study

This study has several limitations. First, the small number of included patients may imply that our results are only applicable to the studied patients, with the need to confirm our findings in larger populations. However, the individual kinetics analysis characteristics showed a similar pattern for all the 5 PH patients, strengthening the results obtained. Moreover, the cardiovascular responses upon exercise onset of the healthy control group were consistent with the expected physiological response to metabolic changes, making it very unlikely that the observed phenomenon could be due to an artifact. Second, it may be that the slower kinetics could have been explained by a lower workload. In fact, this is unlikely as previous physiological study showed that the cardiovascular response is generally faster for lower workload [2] and moreover as the different workload corresponded to similar HR increase in proportion to theoretical HR reserve. Third, the baseline and steady-state CO measurements by thermodilution (CO_TD_) and uncorrected MF [12] (CO_MF_) in PH patients showed differences that might have consequences on data interpretation and confirmed that MF is not accurate in determining absolute CO values without calibration against a reference method [12, 44]. In fact, a correction factor was not imposed to the MF data because we aimed at analyzing kinetics. Under these circumstances, MF has shown to be precise and accurate enough without such a procedure. Nevertheless, the calculated correction factor would have been similar to those previously published for the MF technology [11, 12, 14]. Fourth, characteristics of control subjects differed from the PH patients in several aspects that may have influenced the results. For example, it was shown that kinetics of VO_2_ is slowed as a function of age. However, the kinetics blunting in the included PH patients exceeded by far what could have been expected from normal ageing [45, 46]. Finally, it is likely that results could be different when obtained in upright position where the cardiovascular adjustments were shown to differ from supine position in healthy humans. Nevertheless, RHC is performed in the supine position, so the present study corresponds to the actual clinical setting of hemodynamic evaluation in the catheterization laboratory.

## 5. Conclusions

To our knowledge, this is the first description of beat-by-beat cardiovascular adjustments upon exercise onset in patients with severe precapillary PH. The overall kinetics parameter of HR, SV, and CO appeared slower in PH patients than that in healthy controls. Moreover, we documented a transitory drop in cardiac output upon exercise onset due to a sudden SV reduction, with the CO adaptation to the increased metabolic demand being assumed by HR increases. A larger study is warranted in order to confirm these results and explore their clinical signification.

## Figures and Tables

**Table 1:** Characteristics of the study population. BMI: body mass index.

	Control subjects	PH patients
*n*	10	5
Age, yrs	24.0 ± 3.2	46.8 ± 8.6
Gender F/M	0/10	3/2
Size, cm	179.1 ± 4.3	169.0 ± 8.8
Weight, kg	76.5 ± 5.6	76.4 ± 20.1
BMI, kg.m^−2^	23.8 ± 1.1	26.6 ± 6.0

**Table 2:** Baseline and steady-state exercise hemodynamics of the study population. RAP, right atrial pressure; mPAP, pulmonary artery mean pressure; PAWP, pulmonary artery wedge pressure; MAP, mean systemic pressure; SpO_2_, arterial oxygen saturation by pulsoxymetry; CO_TD_, cardiac output determined by thermodilution; CO_MF_, cardiac output determined by Modelflow; SV_MF_, stroke volume determined by Modelflow; HR_MF_, heart rate determined by Modelflow; PVR, pulmonary vascular resistances; SVR: systemic vascular resistances.

	Baseline (rest)		Effort (steady state)	
	Patients (*n* = 5)	Controls (*n* = 10)	Patients (*n* = 5)	Controls (*n* = 10)
RAP, mmHg	5.4 ± 3.2	—	—	—
mPAP, mmHg	38.8 ± 12.0	—	50.2 ± 19.4	—
PAWP, mmHg	9.4 ± 2.4	—	16.6 ± 9.3	—
MAP, mmHg	86.9 ± 1.2	72.5 ± 1.8	96.8 ± 1.1	76.3 ± 0.8
SpO_2_, %	95.4 ± 3.3	97.2 ± 0.6	95.0 ± 4.3	97.4 ± 0.7
CO_TD_, l·min^−1^	6.3 ± 3.0	—	7.2 ± 2.4	—
CO_MF_, l·min^−1^	8.4 ± 0.1	6.4 ± 0.3	9.5 ± 0.2	10.3 ± 0.2
SV_MF_, ml	94.8 ± 1.9	99.1 ± 2.7	95.3 ± 1.4	110.7 ± 1.7
HR_MF_, min^−1^	89.8 ± 1.1	64.8 ± 2.9	106.1 ± 0.9	92.9 ± 1.1
PVR, WU	5.7 ± 4.0	—	5.4 ± 3.7	—
SVR, WU	10.4 ± 0.2	11.4 ± 0.7	10.2 ± 0.2	7.4 ± 0.2

**Table 3:** Parameters describing the heart rate (HR), stroke volume (SV), and cardiac output (CO) kinetics upon exercise onset for PH patients (*n* = 5) and control subjects (*n* = 10). *b*, baseline mean value as determined by model 1; min, minimal value detected by models 2 and 3; *A*_1_, amplitude of the parameter increase; *τ*_1_, time constant; *d*_1_, time delay of the exponential; *t*_min_, time of minimal value determined for models 2 and 3; MRT, mean response time; ss, steady-state exercise mean value as determined by the model. ^*∗*^Analysis with model 2 was possible only for 8 control subjects.

		PH (*n* = 5)					
		*b* or min	*A*_1_	*τ*_1_ (s)	*d*_1_ or *t*_min_ (s)	MRT (s)	ss
HR (min^−1^)	Model 1	87.7 ± 14.5	20.0 ± 15.9	39.3 ± 24.2	−2.3 ± 12.2	37.1 ± 27.7	106.4 ± 23.5
	Model 2	91.9 ± 18.4	16.1 ± 10.6	50.3 ± 39.0	12.7 ± 11.2	63.0 ± 30.2	106.4 ± 23.5
	Model 3	88.6 ± 15.7	18.6 ± 13.0	194.2 ± 336.4	10.6 ± 12.0	204.8 ± 347.9	106.5 ± 23.5

SV (ml)	Model 1	90.6 ± 32.1	48.3 ± 89.2	372.8 ± 787.7	60.4 ± 83.1	433.2 ± 868.7	97.6 ± 38.8
	Model 2	80.0 ± 34.1	17.2 ± 215	13.4 ± 17.4	13.8 ± 7.3	27.2 ± 24.7	97.7 ± 38.7
	Model 3	76.2 ± 32.5	20.6 ± 24.7	10.4 ± 12.6	12.5 ± 8.5	22.9 ± 20.8	97.7 ± 38.7

CO (l·min^−1^)	Model 1	8.1 ± 3.1	1.9 ± 1.2	88.3 ± 79.0	45.2 ± 65.2	133.5 ± 130.7	9.8 ± 2.5
	Model 2	7.5 ± 3.2	2.2 ± 1.7	42.7 ± 38.2	11.9 ± 9.5	54.7 ± 38.2	9.8 ± 2.5
	Model 3	7.0 ± 3.2	2.7 ± 1.9	27.2 ± 22.4	10.8 ± 10.3	38.0 ± 23.5	9.8 ± 2.5

		Controls (*n* = 10)					
		*b* or min	*A*_1_	*τ*_1_ (s)	*d*_1_ or *t*_min_ (s)	MRT (s)	ss

HR (min^−1^)	Model 1	60.7 ± 11.1	32.3 ± 8.6	16.2 ± 6.9	−7.4 ± 3.1	8.8 ± 7.3	93.7 ± 8.2
	Model 2^**∗**^	76.6 ± 11.0	17.0 ± 3.7	44.2 ± 86.0	7.9 ± 10.1	52.1 ± 92.8	93.2 ± 9.0
	Model 3	74.2 ± 12.2	19.0 ± 6.7	19.4 ± 14.7	3.9 ± 7.1	23.3 ± 21.7	93.7 ± 8.2

SV (ml)	Model 1	98.5 ± 17.1	16.5 ± 6.8	68.3 ± 82.6	5.7 ± 10.6	73.9 ± 84.8	113.3 ± 17.7
	Model 2^**∗**^	98.8 ± 19.1	13.5 ± 3.8	22.3 ± 18.2	16.4 ± 21.5	38.7 ± 28.8	112.3 ± 19.8
	Model 3	94.0 ± 17.6	18.8 ± 6.5	32.6 ± 28.4	3.9 ± 6.2	36.5 ± 28.2	113.3 ± 17.7

CO (l·min^−1^)	Model 1	6.0 ± 1.5	4.4 ± 1.5	26.3 ± 12.7	−6.2 ± 3.9	20.2 ± 13.4	10.6 ± 2.1
	Model 2^**∗**^	7.7 ± 2.5	2.8 ± 0.9	30.2 ± 22.0	9.0 ± 17.7	39.2 ± 27.6	10.5 ± 2.3
	Model 3	7.4 ± 2.2	3.1 ± 0.8	31.9 ± 18.8	1.1 ± 0.8	32.9 ± 18.6	10.6 ± 2.1
